# EDDAMAP: efficient data-dependent approach for monitoring asymptomatic patient

**DOI:** 10.1186/s12911-020-01258-z

**Published:** 2020-09-29

**Authors:** Daniel Adu-Gyamfi, Fengli Zhang, Albert Kofi Kwansah Ansah

**Affiliations:** 1 Department of Computer Science and Informatics, University of Energy and Natural Resources, Sunyani, P O Box 214 Ghana; 2 School of Information and Software Engineering, University of Electronic Science and Technology of China, Chengdu, 610054 China; 3 Department of Computer Science and Engineering, University of Mines and Technology, Tarkwa, P O Box 237 Ghana

**Keywords:** Asymptomatic patient, Data-dependent technique, Health decision-support system, Pandemic, Patient monitoring system, Place of interests, Preventive intervention, Public health, Stay place, Trajectory data mining

## Abstract

**Background:**

A pandemic affects healthcare delivery and consequently leads to socioeconomic complications. During a pandemic, a community where there lives an asymptomatic patient (*AP*) becomes a potential endemic zone. Assuming we want to monitor the travel and/or activity of an *AP* in a community where there is a pandemic. Presently, most monitoring algorithms are relatively less efficient to find a suitable solution as they overlook the continuous mobility instances and activities of the *AP* over time. Conversely, this paper proposes an EDDAMAP as a compelling data-dependent technique and/or algorithm towards efficient continuous monitoring of the travel and/or activity of an *AP*.

**Methods:**

In this paper, it is assumed that an *AP* is infected with a contagious disease in which the EDDAMAP technique exploits a GPS-enabled mobile device by tagging it to the *AP* along with its travel within a community. The technique further examines the Spatio-temporal trajectory of the *AP* to infer its spatial time-bounded activity. The technique aims to learn the travels of the *AP* and correlates them to its activities to derive some classes of point of interests (POIs) in a location. Further, the technique explores the natural occurring POIs via modelling to identify some regular stay places (SP) and present them as endemic zones. The technique adopts concurrent object feature localization and recognition, branch and bound formalism and graph theory to cater for the worst error-guaranteed approximation to obtain a valid and efficient query solution and also experiments with a real-world GeoLife dataset to confirm its performance.

**Results:**

The EDDAMAP technique proofs a compelling technique towards efficient monitoring of an *AP* in case of a pandemic.

**Conclusions:**

The EDDAMAP technique will promote the discovery of endemic zones and hence some public healthcare facilities can rely on it to facilitate the design of patient monitoring system applications to curtail a global pandemic.

## Background

In recent decades, the attention of the public healthcare setting is drawn to the eradication of a global pandemic. During a pandemic, health officials develop an interest in knowing the health status of patients. By so doing, the patients are made to undergo health monitoring [1] by either visiting a health facility or using a GPS-enabled mobile health information system, etc. Usually, the monitoring is done by recording the whereabouts of the patients as well as their routine activities [2, 3], such as a visit to health centre, theatre, tourist attraction, market, shopping mall, restaurants, and other public facilities and functions. Moreover, nowadays, the activity records [4, 5] of patients have become very useful in public healthcare settings for management purposes. This is because the activity records can be used for planning and decision making. For instance, inferencing the whereabouts of patients [6] by considering their activity records in real-time will considerably contribute to an informed decision. And thus the public healthcare facilities can derive some key information from the activity records to support management decisions on the derail of the spread of chronic diseases that can cause a pandemic. Researches show that there is an increasing number of patients with chronic health conditions over the years [7]. Therefore, managing chronic diseases has considerably become a global issue and continues to place pressure on patients in particular and the healthcare setting as well as the society in general [8]. Let’s take a look at an asymptomatic patient (*AP*) as an example. The *AP* is usually diagnosed with a chronic disease but has no noticeable symptoms and for that matter, it appears equally as active as any healthy individual. Therefore, it becomes quite a challenge and/or not ordinary to recognise the *AP* (i.e. without adequate health or laboratory test and/or a medical examination) so that ordinary people can isolate themselves to avoid close contact (i.e. a mechanism to prevent infestation). Despite the health education that is provided to the *AP*, it continues as a threat of the spread of contagious diseases. In this paper, our research motivation is based on the advances in patients health monitoring strategies [4] which make it common for some individuals to wear clothes that are equipped with sensors and/or global position system devices to check their health status [8] on the run (i.e. relatively at any place and everywhere when travelling and/or moving from one place to another). Nonetheless, researches show that the performance of existing monitoring systems continues to deteriorate over time, and this is due to some extent the use of inappropriate and/or under-explored design techniques [4] and the challenges with the tracking of continuous mobility instances and activities of outdoor mobile objects. As a result, it is prudent for researchers to develop some compelling techniques and/or algorithms to support the design, development and management of health information systems that will generally focus on monitoring outdoor mobile patients to curtail a global pandemic. This paper regards the foregoing problem as a maximise range-sum problem [9]. This is because, in spatial databases, the concept of finding the spatial position of objects concerning their location and time (e.g. spatial data stream) involves the fact that the positions of mobile objects need to be recorded over a range in a time frame. For example, Amagata and Hara [9] proposed the G2 algorithm as a maximised range-sum technique for monitoring spatial data streams. The G2 algorithm refers to a Graph in Grid index and consequently addresses the Maximizing Range Sum (MaxRS) monitoring problem [9]. The G2 algorithm is used to update the spatial dynamics of moving objects database incrementally, and thus with respect to time. In this paper, we seek to propose an algorithm and try to compare it with the existing G2 algorithm, to particularly enable us to attest to the validity and performance of our algorithm which is intended for monitoring the continuous mobility instances and the activities of an *AP* in case of a pandemic. Comparatively, the contribution of our proposed technique and/or algorithm to that of the G2 algorithm includes the continuous monitoring instances incorporated into the traditional moving object database. There seems to be a pressing issue when dealing with real-time monitoring of continuous moving objects, with which the existing spatial query methods and/or algorithms can only cater for static objects and/or instantaneous monitoring of moving objects. For that matter, they are not scalable to continuous streaming environments and hence cannot relatively offer a suitable solution. As a way forward, this paper exploits a compelling technique and/or algorithm that is purposely used for the efficient monitoring of continuous MaxRS. Moreover, our future work will consider much more existing related algorithms that are key on temporal attributes, and not only instantaneous but continuous moving object database scenarios and make some comparisons to improve on the performance validation of our proposed algorithm. Even though, when you compare our technique to other existing ones, there is much more resemblance in terms of the methodology. For instance, aside from the G2 algorithm, there are some other proposed algorithms in references [10] and [11] which are not compared due to the resource constraint of the present study. In this paper, however, our focus is to evaluate the validity of the proposed technique and/or algorithm and reserve a deeper comparison of it with the other existing algorithms as future work. Moreover, our proposed technique relies on the analysis of Spatio-temporal trajectory pattern where the core mandate is to detect the point of interest (i.e. not necessarily the point being a hotspot).

**Fig. 1:**
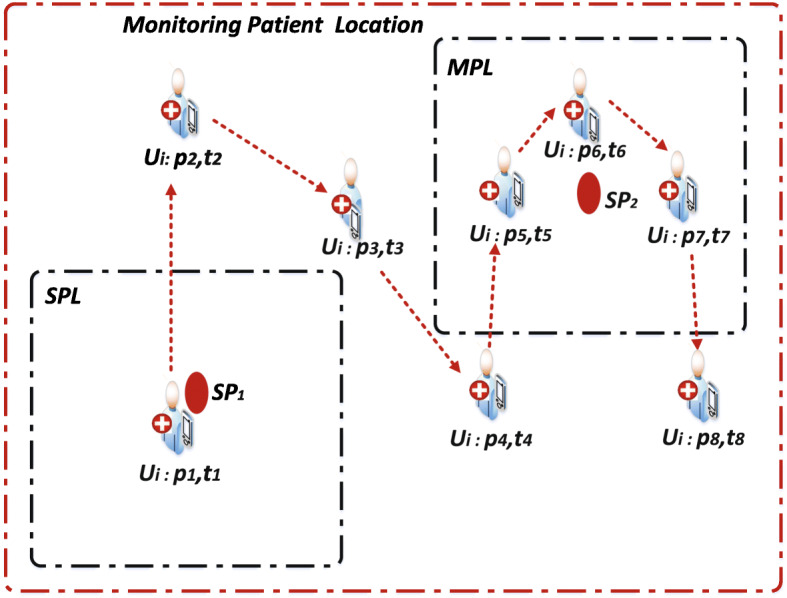
Category of Stay place (*SP*). Showing *SP*_1_: single-point location (*SPL*), and *SP*_2_: multi-point location (*MPL*) of patient based on *POIs*. *Ui* represents user identifier for *AP*, *p* and *t* denote the spatial position (*POIs*) and time-stamp respectively

On the other hand, Zhang *et al* [10] proposed an algorithm which depends on Spatio-temporal clustering analysis and that is based on analysis of hotspot (i.e. point not necessarily a regular interest of the objects). And thus where the objects are always clustered in a region in a time frame. This kind of approach is a better fit for text classification and analysis and thus based on the traditional range queries, and other queries that use similar methodology such as the range query, skyline, k-nearest neighbour, reverse nearest-neighbour and so forth. It is worth noting that, these aforementioned approaches consider the number of object points and/or higher dimensionality objects related to a specified region (i.e. hotspot) [12]. Moreover, pattern and clustering analyses are considered important theories and/or techniques in trajectory data mining and they can apply to construct the behaviour dynamics of moving objects. Furthermore, aside from finding the semantic context and association rules that exist in trajectory data, Qiu *et al* [11] developed a generalized framework to identify the various activities of users about their trajectory records. The approach applies to analyse the everyday lifestyle of mobile users which is far-reaching to determining instantaneous mobility conditions of outdoor mobile users. The foregoing approach is very similar to our work, but the difference may lie on where our approach is centred on the continuous mobility instances of outdoor mobile users and their activities along with their travel. Furthermore, the approaches utilise a sample of a real-world GeoLife dataset produced by the Microsoft Asia Beijing for the validation of the techniques and/or algorithms. Moreover, the Zheng *et al* [13] technique considers a user behaviour prediction via collaborative filtering strategy and that is based on ranking the user’s preference. Also, it does not try to find the accurate prediction value for the user’s activities in a location, but it rather approximates the prediction value based on user preferences, and thus by making it relevant in practice. Furthermore, their study obtains user preferences based on the locations and activities history by ranking the locations and activities of the user for service recommendation purposes. However, using human labellers (i.e. labelling is done by a human being) to parse the user-generated comments whenever a user is found in a certain location to get the activity labels seems not an ideal concept to explore. Moreover, the user comments are in text format and automatically the activities of the user can be detected based on text classification and analysis. Comparatively, rather than relying on the activity history in a specific location, our proposed technique is making use of the specific time and the duration or time frame that a user spends in a location to continuously determine the possible activity and/or behaviour of the outdoor mobile asymptomatic patient (i.e. emulated as an ordinary mobile user). Thus knowing the location of the asymptomatic patient (i.e. a user) through the location coordinates in real-time and that of the specific time and duration used on travelling and/or visiting the location can provide relevant knowledge to predict its behaviour, rather than depending solely on the posted text comments [13] which may potentially be misleading due to issues with privacy, etc. Similarly, finding and/or predicting the exact location of the outdoor mobile user and/or asymptomatic patient will practically not be very necessary and hence the location estimation is usually approximated based on user preferential value which is considered as a user tolerance. Our proposed technique considers a correlation analysis of the location and activity of the user. This approach can reveal some relevant knowledge on the behaviour of the user and thus making it useful in the design of state-of-the-art outdoor mobile recommendation systems. Besides, Smyth [4] conducts a comparative study on sensors and real laboratory data and consequentially reveal that it is much easier to predict the health status of patients via sensor data. Also, El-Sappagh el al [7] prove how easier it is to track the fitness of patients using sensor data. Notwithstanding, Or *et al* [14] propose that most of the modern health information systems have under-explored design technique challenges [5]. Moreover, Rajkumar *et al* [15] propose an improved software intelligent system towards enhancing the performance of a hearing impairment system to provide a solution to audiological problems. Whereas, Singh *et al* [16] introduce a deep learning model towards human activity recognition such that without prior knowledge the model can be used to classify human activities. However, Singh *et al* model [16] will heuristically not be suitable to deploy practically to investigate a continuous moving outdoor user such as an *AP*. For instance, it is important to obtain a prior knowledge on the initial health status of the *AP* follow by monitoring the activities of the *AP* as the stance of the present paper. Furthermore, Nogueira *et al* [5] propose a biofeedback system technology that can be used to evaluate the quality of life of patients. Whereas Crepaldi *et al* [1] evaluate health information systems based on software engineering metrics by emphasising on the system methods and designs and further propose that a lot of the existing approaches for managing the health records of patients are not efficient as a result of overuse of resources. Wu *et al* [17] identify two peculiar issues when dealing with monitoring the mobility of objects. The first issue considers how to correlate the spatial and temporal information about the location of the objects to enhance predictions. The second issue considers how to develop effective and efficient large scale practical-oriented predictive systems e.g. health information decision support systems. Considering these two aforementioned issues, it is quite harder to find a readily available solution. However, once again this paper strongly derives motivation from the ubiquitous built-in Global Positioning System (GPS) mobile devices and/or applications [18]. Using GPS-enabled mobile devices and/or applications coupled with other wireless technologies can improve the solution to monitoring problems. This is because the GPS-based devices can acquire the position and/or location data of an outdoor mobile user on the run [19]. Similarly, it is relatively inexpensive to find the geographical location, the spatial position, the time stamp and the place of interests (POIs) of patients (or outdoor mobile users) by exploiting a GPS-enabled mobile application. Furthermore, monitoring an outdoor mobile patient can be defined as the continuous (or periodic) measurement and analysis of an outdoor mobile patient bio-signals from a distance by employing mobile computing, wireless communications, and networking technologies [20]. Suppose that we want to monitor the various places in a specific community where the *AP* will visit in future. Particularly, let’s try to reveal those places where the *AP* will mostly stay. It is not trivial to achieve a solution to this problem. For instance, a continuous moving *AP* can exhibit some dynamics and/or varying degree of interests with regards to its behaviour. This property contributes to the basic functional attributes of almost every continuous moving object database. Therefore, it can be emphasised that the behaviour of the *AP* is subjected to location influences by either inherent or acquire properties of the location. Therefore, tagging the *AP* with a particular interest and doing so in an incremental fashion and continuous-time intervals (i.e. streaming data instances) becomes a challenging task. This is because the process will require excessive computation [19] and a lot of software and hardware resources. Nonetheless, with the aid of ubiquitous GPS-enabled mobile devices and available methods of computing the position and location of objects, the task can relatively be executed efficiently with less troubles. Therefore, among the benefits of carrying out this relevant task is to reveal the whereabouts of *AP* in real-time to facilitate the derail of a global pandemic. To reiterate, the focus of this paper is mainly to propose EDDAMAP which is referred to as an Efficient Data-Dependent Approach for Monitoring Asymptomatic Patient. The objective of EDDAMAP is to facilitate to detect the stay place (*SP*) of *AP* to ensure proper and efficient monitoring of *AP* within a community. We consider the *POIs* based on the Spatio-temporal trajectories [21] of the *AP*. Thus when an *AP* is visiting some interesting places and engaging in certain activities in a community within some time intervals. It is worth noting that there exist two categories of *SP* [21] when dealing with object monitoring. These are single-point location (*SPL*) and multi-point location (*MPL*). The *SPL* refers to a location where *AP* has visited and spent an inordinate amount of time, and this is illustrated in Fig. 1. Whereas *MPL* refers to a location where *AP* has visited and spent relatively a short time and this is also illustrated in Fig. 1. These two categories of *SP* significantly contribute to trajectory analyses towards objects location monitoring and prediction problems [21]. Given a set of *POIs*, unit weight and user specified-sized rectangle *r*, we aim to find an enclosing *r* with maximised weighted-sum of *POIs* and locate a centroid as *SP*. Assuming a prior knowledge of *AP*, we employ similar algorithmic design from work [9] and concurrent localisation and recognition, branch and bound formalism and multi-object instance methods from work [22] by considering the *AP* and its current time-stamp position in a location under general monitoring. The Spatio-temporal trajectories of the *AP* that consist of *POIs* are generated in a continuous-time interval to support the design of grid-based index data structure and algorithm towards achieving a valid and efficient solution for detecting an *SP*. The *SP* information can serve as relevant knowledge to the public healthcare facilities to inform the potentials of an *AP* on spreading contagious diseases. This paper is particularly relevant to the public healthcare settings and computing communities as it provides an overview of the technicalities involved in monitoring a mobile patient and also highlights issues related to the design of monitoring systems. The propose EDDAMAP technique and/or algorithm can be utilised by the healthcare settings to improve the features, design and development of prospective health information system applications for monitoring an outdoor mobile patient in case of a pandemic. On the other hand, the computing community and the related fields of study will gain a broader view of the novel techniques for the design and development of large scale health information system applications on outdoor mobile patients monitoring.

In summary, this paper seeks to derive a compelling monitoring technique towards advancing the health information technology industry by proposing a trajectory data mining oriented design technique and/or algorithm for the continuous monitoring of the travel, activities and health of an outdoor mobile asymptomatic patient in case of a pandemic. Trajectory data mining and/or Spatio-temporal trajectory mining is not entirely a new research area in computing. The domain has been explored in the recent past to find a solution to several real-world and multi-facet problems [23–27]. Thus, the proposed technique and/or algorithm will improve the design and management of state-of-the-art outdoor mobile patient monitoring system applications. The experiment result evaluation validates the proposed EDDAMAP technique as robust and easy to implement and can also support the design of existing patient health monitoring systems. Further, the public healthcare settings can rely on the technique to improve patient monitoring system applications as well as to make an informed decision on a global pandemic.

The remainder of the paper is as follows. The method underlying the propose EDDAMAP technique is detailed in “Developing the algorithm” section. In the “Evaluating the algorithm” section, we present a graph of the simulation results of the propose EDDAMAP algorithm and the result discussions with possible improvement. Finally, in the “Conclusions” section, we present the conclusions of the paper with some future work.

## Method

### Developing the algorithm

#### Trajectory

First of all, we define trajectory as a sequence of tuple given by *P* = {(*x*_1_,*y*_1_,*ta*_1_,*ts*_1_),...,(*x*_*k*_,*y*_*k*_,*ta*_*k*_,*ts*_*k*_)}. Such that *ta*_1_<*ta*_2_<,..., <*ta*_*k*_ and *ts*_1_<*ts*_2_<,..., <*ts*_*k*_. Where *P* is referred to as any spatial position of outdoor mobile user or object, *x* and *y* represent the location coordinates, *ta* and *ts* denote the respective arrival time and stay time of a user who is in a specified location. It is essential to also note that *P* is subjected to a unit positive weight including other location features such as altitude. However, the altitude is insignificant so far as trajectory analysis is concerned [21].In this paper, we refer to the Spatio-temporal trajectory as the time-bounded spatial activity of continuous moving outdoor mobile user such as *AP*.

#### Trajectory monitoring instances

Assume that *P*(*n*) has countably-infinite *POIs*. If *p*(*i*) is any identified *POIs* then it follows that *p*(*i*)={*x*,*y*,*w*}. Such that *p*(*i*)∗*w*∈*R*^+^ and *p*(*i*)∈*P*(*n*). Where *x* and *y* are spatial position coordinates and *w* is a positive weight which is defined by features of the monitored location where the outdoor mobile user (e.g. *AP*) is positioned. Our question is how to find stay place *SP* of an *AP* who is loitering around any outdoor location. Motivation is being derived by computing for the most visited and stay place of the *AP* within the monitored location with the aid of GPS data on the *AP*. Hence we regard the monitored location as a community where the *AP* will live, stay and/or travel and at the same time engaging in various routine activities such as dining, shopping. spotting, visiting, adventuring, and a lot more. Suppose that *m* represents a search space and *n* represents an entire monitored location. Using the Eq. 1 we can obtain *p*(*i*) such that *i* = 1,...,*m* and *m* <*n*. This will imply that if *m* = *n* then no relevant task to execute due to the search space being equal to the entire monitoring location. Moreover, if *m* >*n* then the task is not practical to merit execution.

1 $$\begin{array}{@{}rcl@{}} P(i)w = \Sigma P(i) * w \end{array} $$

**Fig. 2:**
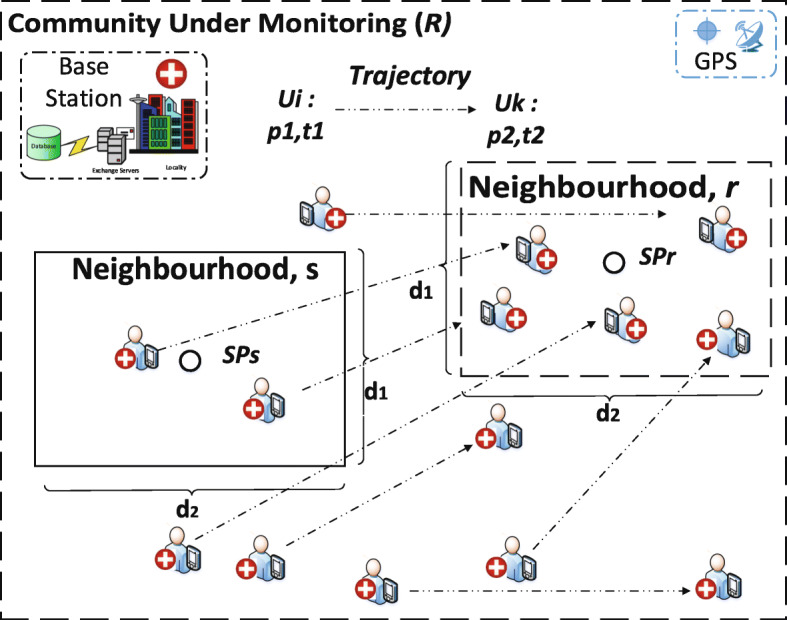
A derived stay place *SP* showing the trajectory of asymptomatic patient *AP* based on *POIs*. *SPs* and *SPr* are obsolete and update (or new) centroid respectively which indicate any *SP*. Base station represents the centre that is responsible for the acquisition, storage and management of the GPS data that are acquired via the monitoring device and/or system application of the *AP*

Suppose that *R* is a community and *r* is a specific location (e.g. neighbourhood) in *R* such that *r*∈*R*. Heuristically, we can monitor *r* continuously to obtain any *POIs* which will represent the point of interest of the *AP*. Let *p*(*i*)∈*p*(*n*) denote weighted-sum of the *POIs*. To ensure that any *p*(*i*)^′^*s* are maximised in *r* we heuristically establish the objective function in Eq. 2 to handle such query. Where *p*(*i*)(w) denotes any identified weighted *POIs*. As a result, monitoring the *r*(*w*) will be the same as the *SP*. The process is simplified as shown in Fig. 2.

2 $$\begin{array}{@{}rcl@{}} r(w) = \arg \max_{i\in n \cap R} p(i)* w \end{array} $$

#### Concurrent localisation and recognition of *POIs*

This section presents how to locate, recognise and instantaneously monitor an *AP* when it is travelling and/or visiting interesting places in a given community such that we can explore the *POIs* to discover an *SP*. Of course, the *SP* is going to serve as a source of relevant information for the health setting to support decision making, because the *AP* can be a potential spreader of contagious disease and for that matter can cause a pandemic. Assuming there are *n* location features and *p*_(*n*)_ models which define the labels of the location. We adopt concurrent localization and recognition technique [22] to find a bounding rectangle *r* (i.e. achieving localization of the *AP*) that will enclose the various *POIs*. Also, we try to identify a model *p*_(*i*)_ that will maximise a prediction score *L* for the *POIs* (i.e. achieving recognition of the *AP*). When predicting the *POIs*, there will be *L*_(*i*)_ score (i.e. obtaining any partial score) which can either be an output of support-vector machine (*SVM*) classifier (i.e. achieving identification of the *POIs*) or tree-based index (i.e. achieving categorisation of the *POIs*). Alternatively, the prediction score which is denoted as *L* can be obtained by accumulating the partial scores denoted as *L*_(*i*)_ which are obtained from the various *POIs*. For that matter, we proceed by computing the prediction score *L* in an incremental fashion, and thus whenever a new *POIs* is discovered and/or a *POIs* is obsoleted and/or discarded. Assume that an anticipate prediction score is *p*_(*k*)_ when searching for a subsequent *POIs* (i.e. achieving evolution). We compute a *p*_(*k*)_ by creating an upper bound of the monitored location. Therefore, we employ that of the branch and bound formalism and/or technique [22,28] which can handle worst error-guaranteed approximation to obtain an arg max*kp*_(*k*)_ by considering all the *POIs* data. Furthermore, the plane-sweep mechanism [22,28] is adopted as a suitable strategy to create an interval that will capture all the *POIs* data. Thereafter, we iterate the *POIs* within the location to split the location interval into two disjoints sets. Using the two disjoint intervals, we apply bounding strategy to decide on the next location for the *POIs*. As a result, the iteration is terminated where *k* becomes a singleton. We then consider that stage or state of the iteration as the point of optimal location for the *POIs*.

Expressing *n* location features given by the triplets, *F*=: {(*ax*_*k*_,*by*_*k*_,*cL*_*k*_)|*k*:1,...,*n*} as any input, where *x* and *y* are the location coordinates and *L* is the location feature which is an M-dimensional vector. Whereas *a*, *b*, and *c* are constant coefficients and *L*_(*i*)_ denotes *i*−*th* element of *L*_(*k*)_ where *i*<*k*. We can find the partial scores *L*_(*i*)_ with which the various *POIs* will contribute to the total prediction score *L* and establish the optimization objective in Eq. 3. It follows from Eq. 2 that *F*_(*r*)_ will represent the *POIs* that are spatially bounded by the *r*. Therefore, computing for the $\overrightarrow {L_{(k)}}$, it implies that the *POIs* can equally be obtained. This will imply that we can have more than one neighbourhoods within one community such that the *AP* can be continuously monitored whenever it is travelling or moving from one neighbourhood to another in the community and by so doing visiting places of interests in the community.

3 $$ \arg \max_{r,i}\sum_{k \in F(r)}^{}\overrightarrow {L_{(k)}}(i)   $$

Suppose that we want to train *SVM* classifier for any *POIs*. The *POIs* can be taken as input to evaluate the *SVM* classifier margin by comparing any testing sample to each of any training sample. We can compare the weighted *POIs* that have support vector with any other aggregated partial scores to obtain a set of similarity scores. We can sum up the similarity scores by considering a chosen *SVM* offset values *δ*_*i*_ and then express the margin as the weighted-sum of the *POIs* (i.e. considering all the partial similarity scores). This approach is not far from the one that is used in existing work [22] and thus if not the same. Denoting the $\overrightarrow {\mu _{p}}$ as any vector with support vector weights for the *p*th training set of the *POIs* data, such that *w* ∈*w*_(*k*)_. Assume that the *i*th element of *p*th training *POIs* denotes the weight of the *i*th *SVM*. If *p* is denoted as any identified *POIs*, then *l*_*p*_ and *c*_*p*_ will be the model label and feature score respectively. Therefore, the anticipated partial score can be computed via Eq. 4. It can be noted that h(·) will evaluate to 1 whenever the function is true (i.e. representing a true solution) and then it evaluates to 0 for false (i.e. representing no solution).

4 $$ \overrightarrow{L_{(k)}}(i) = \sum_{p} \in w_{{(k)}}^{} h(l_{p} = i) c_{p} \overrightarrow{\mu_{p}}(i)   $$

#### Branch and bound formalism

Drawing on the concept of the branch and bound formalism [22], we ensure that search space is disintegrated into disjoint subspaces. Through that, we discover the optimal value to represent the *POIs*. Heuristically, we assume *w*_0_ and *w*_1_ as maximum weighted-sum *POIs* and weight of monitored location respectively. Let *tol* denotes the user error-tolerance, we conjecture that *w*_0_≥*w*_1_(1−*tol*). Therefore, there will be a trade-off between query efficiency and *tol*. However, the *tol* can always be fine-tuned such that it will be slightly higher than an actual (or a realistic) error-rate.

##### Branch technique

In this section, we discuss how our algorithm applies the branching operation as given in *algorithm* 1 to find the optimal location as the *POIs*. Let *d* be a subspace, and *d*_1_ and *d*_2_ be any two smaller subspaces. Base on upper bound estimation, and thus by considering the *x* and *y* coordinates of the general monitoring location (i.e. R) and *m* object models, we obtain *Z*_1_ and *Z*_2_ as two subsets of equal-size (i.e. as candidates). We transform the subspace *d* as given by *d*_(*i*)_ =: {(−*x*_(*i*)_,+*x*_(*i*)_),(−*x*_(*i*)_,+*x*_(*i*)_),*m*_(*i*)_}, such that *i* = 1, 2 (i.e. binary) and *y* is split as *d*_1_ (i.e. points in *Z*_1_ are removed from −*y* as well as all *x*_1_).

##### Bound technique

In this section we discuss how our algorithm applies the bounding operation to determine the largest and smallest bounding locations. This can be done by considering a given subspace *d* using the −*x*, +*x*, −*y*, +*y* and *m* parameters. Considering the *x* and *y* extremities, we obtain +*b* (i.e. + *L*_*i*,*j*_) and −*b* (i.e. - *L*_*i*,*j*_) as the largest and smallest rectangle (i.e. location) sizes respectively. Let *a* represent label for object model *m*, such that *a* ∈*m*. Using the dimension of *a*, we consider −*b* and +*b* intervals, and locate a space which includes as many positive weighted *POIs* (i.e. $\overrightarrow {Z_{k}}$(*a*) >0), and thus excluding as many negative weighted *POIs* (i.e. $\overrightarrow {Z_{k}}$(*a*) <0). At this stage, the Eq. 2 can heuristically apply. Considering the *POIs*, if there are more positive values than +*b*, and few negative values than −*b* in the rectangle (i.e. location), then it is assumed that an optimal solution is found. Otherwise, it is irrelevant to compute or search for a new rectangle (i.e. as an optimal or best location for the *POIs*). We can heuristically compute the upper-bound say +*J* via Eq. 5. Similarly, lower-bound say −*J* can be computed via Eq. 6, and thus by adding many negative values, and subtracting many positive values. Both of the +*J* and −*J* are assumed to have object model *a* as a function of subspace *d*.

5 $$ +J_{a}(d)=\sum_{j \in d(+b)} +h\left(\overrightarrow{Z_{k}}(a)\right) + \sum_{j \in d(-b)} -h\left(\overrightarrow{Z_{k}}(a)\right)   $$

6 $$ -J_{a}(d)=\sum_{j \in S(-b)} +h\left(\overrightarrow{Z_{k}}(a)\right) + \sum_{j \in d(+b)} -h\left(\overrightarrow{Z_{k}}(a)\right)   $$

Where +*h*(*x*) and −*h*(*x*) are functions. If *x* is positive then +*h*(*x*) is evaluated. On the other hand, if *x* is negative then −*h*(*x*) is evaluated. Otherwise, the function will evaluate to 0 when *x* is neither positive nor negative. Finally, we compute the overall upper bound +*J* and lower bound −*J* for the subspace, *d* as given in Eqs. 7 and 8. Therefore, any *SVM* classification problems can be trained by adjusting the bounding estimates using offset values, say *δ*_*a*_ of the respective *SVM* classifier via Eq. 7.

7 $$ +J_{a} = J_{a} + \delta_{a}   $$

8 $$ -J_{a} = J_{a} + \delta_{a}   $$

##### Algorithmic pseudocode for *POIs* execution

We construct the algorithmic pseudocode for the *POIs* execution as provided in Algorithm 1.


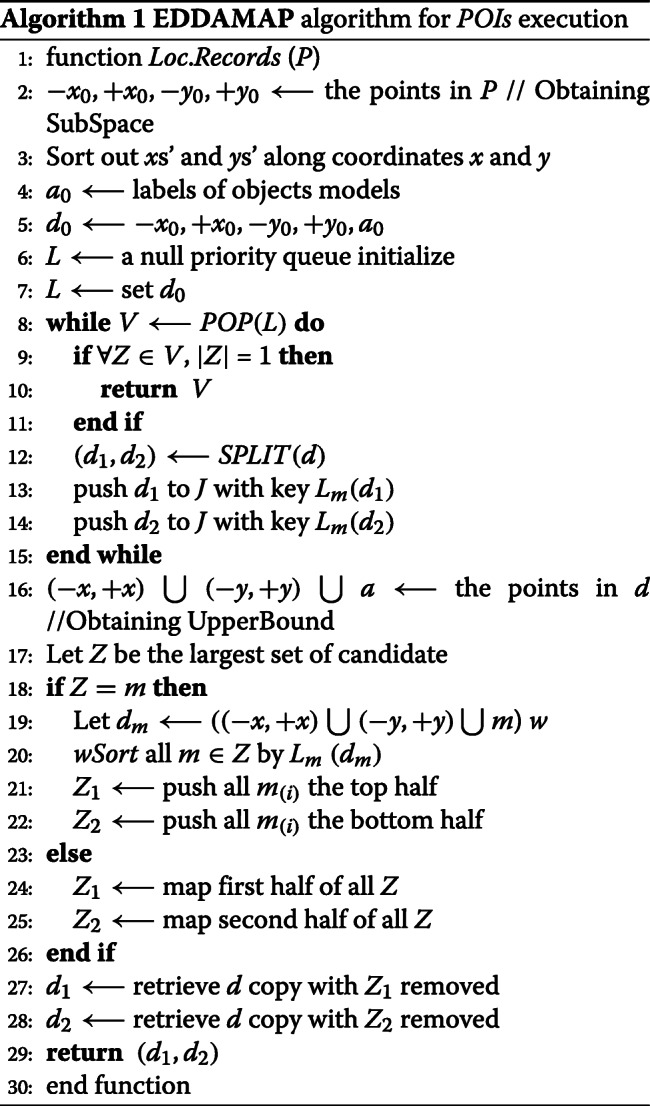


#### Graph analysis and index data structure

Graph theory and network topology [19] are used in various domain of researches to find a solution to complex and dynamic network-related problems. Thus, graph base and topological data mining provide knowledge representation base on graph analysis to construct novel queries for the analysis of complex and dynamic network-related problems. Such as analyses of network connectivity behaviour [29], patterns and impacts of network systems, continuous Spatio-temporal trajectory joins [30], etc. Here we discuss the construction of a generalised index data structure for processing the trajectory of *AP* based on graph analysis.

**Fig. 3:**
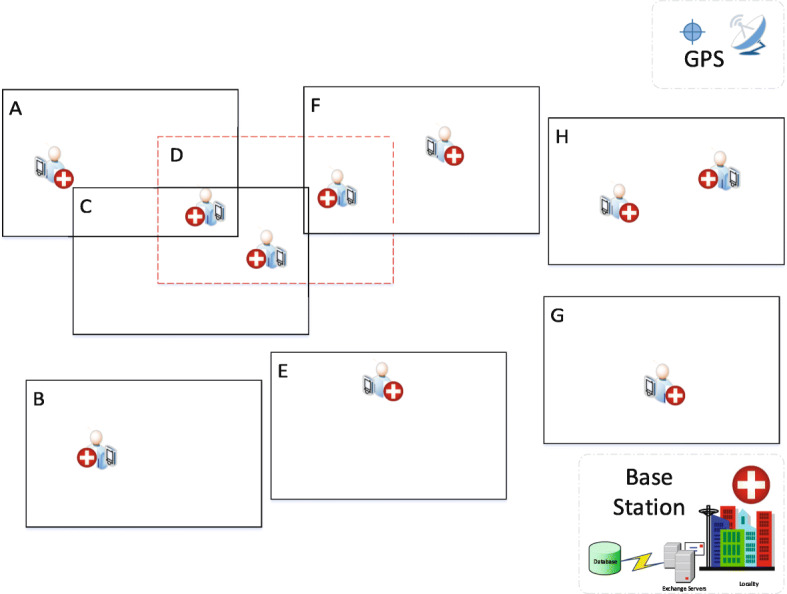
Example of tracking position instance of asymptomatic patient. Showing the various instances of *POIs* in monitoring location or neighbourhood identified by A, B, C, D, E, F, G and H. The neighbourhood D has the maximised weighted-sum of 3 *POIs* and it is classified as the optimal solution (or neighbourhood) to represent *SP* of the patient. Base station ensures the acquisition, storage and management of the monitoring data from the GPS device

Let’s consider Fig. 3 which is an illustration of tracking instances of *POIs*. Assuming there are ten positions instances of the *AP* representing *POIs* enclosed within rectangles *A*,..., *H* (i.e. representing individual places of visit or neighbourhood). As shown in 3, it can be seen that the red broken-line rectangle provides the optimal solution for having three of the total *POIs* as the maximised weighted-sum.

**Fig. 4:**
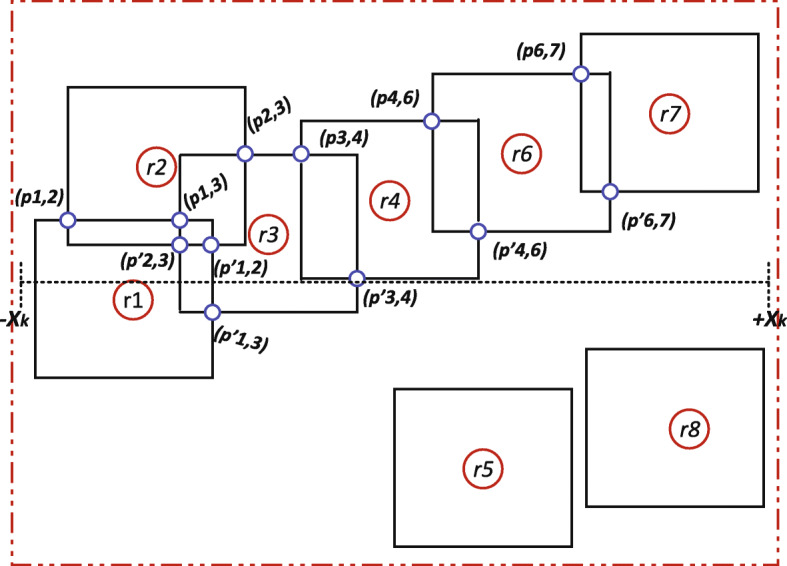
Transformation of *POIs* into rectangles. Showing rectangles *r*_1_,..., *r*_8_ and intersection generated by *p*_*i*_,_*j*_ and $p^{\prime }_{i},_{j}$

**Fig. 5:**
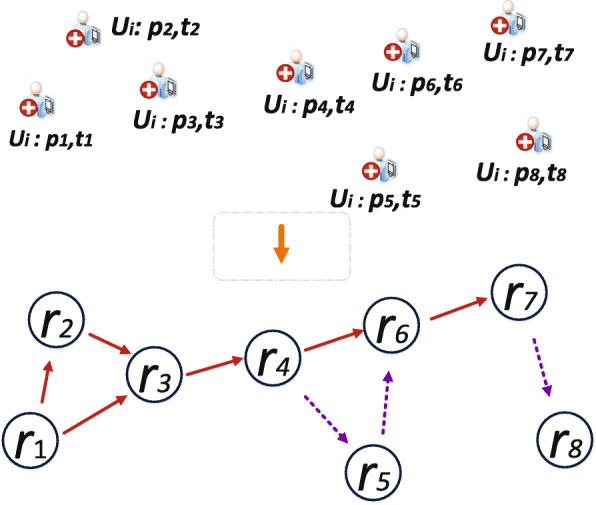
A tree graph of *POIs*. Showing trajectory instances of *AP* based on *POIs*. *Ui* represents user identifier for *AP*, whereas *p* and *t* represent the spatial position (*POIs*) and time-stamp respectively

**Fig. 6:**
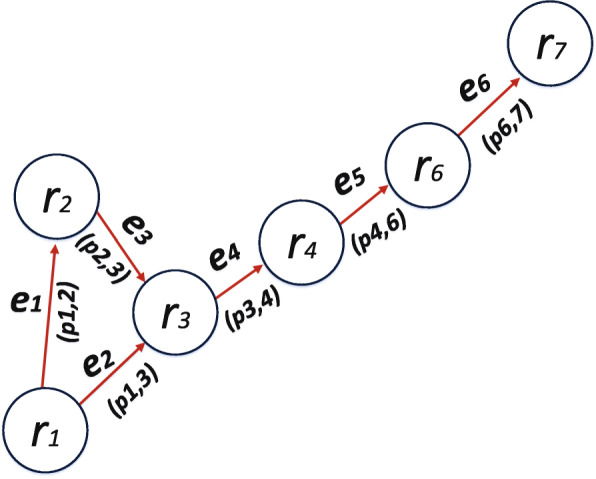
A tree data structure of *POIs*. Showing the core or usual trajectory instances of *AP* based on *POIs*

**Fig. 7:**
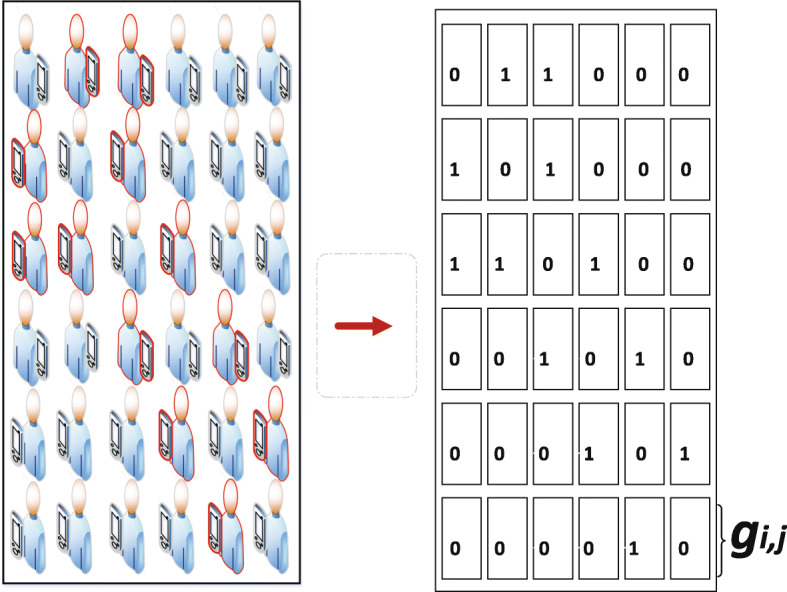
A schematic view of data structure for maintaining any answers for a Patient’s *POIs* query in continuous case

Let *G* = (*V*, *E*) denotes a dynamic graph. Vertices *V* represent the position data leading to the discovery of *POIs*. As shown in Fig. 3, vertices are further transcribed as rectangles. An overlap of two vertices generate edges *E*. Therefore, one-to-one mapping of vertices corresponds to edges. As shown in Fig. 3, we transform the *POIs* into vertices. As a result, we can now construct the dynamics of monitoring *POIs* in continuous time intervals via graph analysis. Following the dynamic process, we can derive the trajectory instances of *AP* based on Fig. 4. Therefore, we construct a tree graph to represent the dynamics of trajectory instances of *AP* [31] based on *POIs* as shown in Fig. 5. Further, we construct a tree data structure by concentrating on the core or usual trajectory of *AP* as shown in Fig. 6. It should be noted that graph *G* = (*V*, *E*) is dynamic, hence it can be reconstructed to reflect the dynamics of mobility. Therefore, if *r*_*i*_, *r*_*j*_∈*V* is generated then by conversion *r*_*i*_ is older than *r*_*j*_. We present the relationship between graph *G* = (*V*, *E*) in Table 1. We then apply the concept of Origin-Destination (OD) matrix to construct a list adjacency matrix. By so doing, it is now possible to design a generalised grid-based index data structure to cater for the continuous evolution of *POIs* leading to the discovery of *SP*. Thereby, we construct a grid-based index data structure as shown in Fig. 7.

**Table 1:** A tabulated relationship of vertices, edges, and next neighbour vertices based on graph *G*

**Vertex**$\left (V_{(r_{i})}\right)$	**Edge (*e***_***i***_**)**	**Next neighbour Vertex**$\left (\text {N}V_{(r_{i})}\right)$
1	e1,e2	2,3
2	e3	3
3	e4	4
4	e6	6
6	e7	7
7	null	null

Drawing on the study of graph theory, three properties can be defined based on Fig. 6 [9]. Firstly, if *r*_*i*_, *r*_*j*_∈*V*, then *p*_*i*_≠*p*_*j*_. Thus, if and only if there exists an overlap between *V*(*r*_*i*_) and *V*(*r*_*j*_). For instance, where *V*(*r*_*i*_) is older than *V*(*r*_*j*_), then (*V*(*r*_*i*_),*V*(*r*_*j*_)) = *r*_*ij*_ = *e*_*i*_∈*E*. Also, if *e*_*i*_≠*e*_*j*_, then *NV*(*r*_*i*_)≠*NV*(*r*_*j*_), making *p*_*i*_ a subspace of *V*(*r*_*i*_). Secondary, if *P* grid cells or spaces contain a set of *p*_*i*_ enclosed in *r*_*i*_, then Eq. (2) is verifiable. It means those obsolete vertices are no longer needed to undergo maintenance as a result of the corresponding edges being held by the existing vertices. Thirdly, if *r*_*i*_∈*V* then vertex *r*_*i*_ is considered as obsolete in circumstances where there are older vertices, and hence any *p*_*i*_ becomes the current position of *AP*. When *AP* moves from one place to another, the position data need to be updated, say *R*^*u*^. Therefore, we *R*^*u*^ compare with all the existing *POIs* to update the gride cell or data structure accordingly. If *m* represents newly generated *POIs* and *n* represents the rate, then the time complexity for the process becomes *O*(*mn*), which is quite high. Therefore, we need to find a more suitable solution to improve on the complexity. Consequently, we modify *G* = (*V*,*E*) as illustrated in Fig. 5 to obtain *G*_(*i*,*j*)_ = (*V*_(*i*,*j*)_,*E*_(*i*,*j*)_) as illustrated in Fig. 6. Thereby, cell *g*_*i*_ or *g*_*j*_ can be maintained in the graph *G*_(*i*,*j*)_. As a result, if *m* new vertices are generated and added to *G*_(*i*,*j*)_, then overlaps need to be checked with the corresponding *g*_*i*_ and *g*_*j*_ to update the grid cell data structure by a factor of *G*^*u*^(*i*). Hence, at this stage, the time complexity will improve from *O*(*mn*) to *O*(*m*) for the total overlap. Therefore, the overall time complexity of the EDDAMAP algorithm becomes *O* (|*G*^*u*^(*i*)|*m*^*u*^,*n*^*u*^), which is very reasonable and a far-reaching efficient solution. Where *m* is the average number of vertices undergoing overlap, and *n* is the average number of overlap. It should be noted that *m*^*u*^<<*m* and *n*^*u*^<<*n*. Hence, the cost of storage becomes *O*(|*V*|+|*E*|) for all *G*_(*i*,*j*)_ = (*V*_(*i*,*j*)_,*E*_(*i*,*j*)_), and for every *r*_*i*_ to maintain *p*_*i*_. Also, the worst space complexity becomes *O*(|*V*|^2^) for *n* rounds of overlap, and that is almost equal to *O*(|*O*|^2^) which is quite impractical to evaluate as worst space [9].

##### Continuous monitoring of *POIs*

Making use of Fig. 7, we can perform real-time monitoring of *POIs* towards achieving an *SP*. This is done by following the trajectory of the *AP* [32,33]. We deduce the maximised weighted-sum of *POIs* and obtain a centroid as *SP*. Knowing the *SP* at some instance, we can compute newer *SP* by understanding the mobility pattern of the *AP* [3,34]. Let *l*(*w*) and $\bar {p}(i)$ represent new *SP* and new *POIs* respectively. Supposing we have an existing cell *g*_*i*,*j*_∈*G*. If $g_{i,j} \dot w \textgreater l(w)$, then all the vertices in *V*_(*i*,*j*)_ (i.e. *R*) do not contain *l*(*w*). Therefore, it is not sufficient to compute the exact solution for *p*_(*i*)_, where *r*_*i*_∈*V*_(*i*,*j*)_. Moreover, supposing cell *g*_*i*,*j*_∈*G*, and assuming $g_{i,j}\dot w \geq l(w)$, where vertices (or rectangle) $r_{i'} \in V_{(i,j)}\phantom {\dot {i}\!}$. If $l(\hat {w}) \textless p(i)w$ then $\phantom {\dot {i}\!}r_{i'}$ does not contain *p*(*i*)*w*. Hence, we do not compute the exact solution for *p*(*i*)*w*. Consequently, Eq. 11 can be used to evaluate the evolving *SP*. Intuitively, we set *k*-th largest weight as threshold upon continuously monitoring the *topk* of the *POIs*. Also, it is important to bound any error-rate based on a defined user-tolerance (*tol*). Heuristically, we establish the optimization function as follows. Furthermore, we construct the algorithmic pseudocode for monitoring the *POIs* as given in Algorithm 2 and Algorithm 3.

9 $$  l(w) = l(w) + \sum_{i}^{k} \bar{p}(i)  $$

10 $$  l(w) = l(w) + \sum_{i}^{k} l(w)  $$

11 $$  g = \arg \max_{g_{i,j} \in G\cap R} g_{i,j}{w}  $$

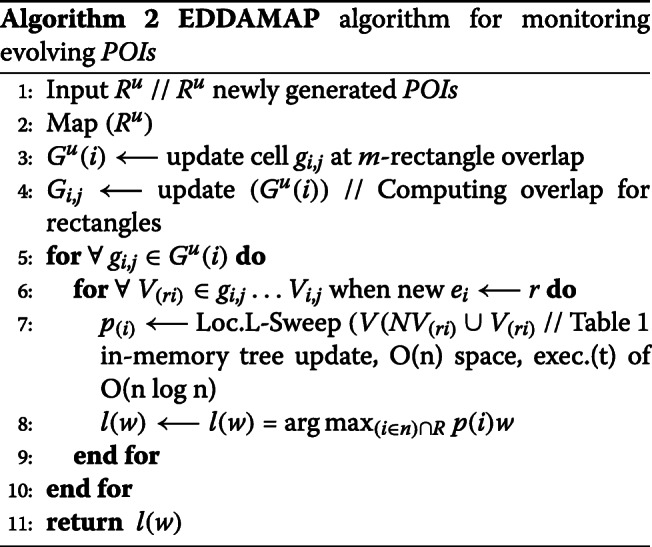



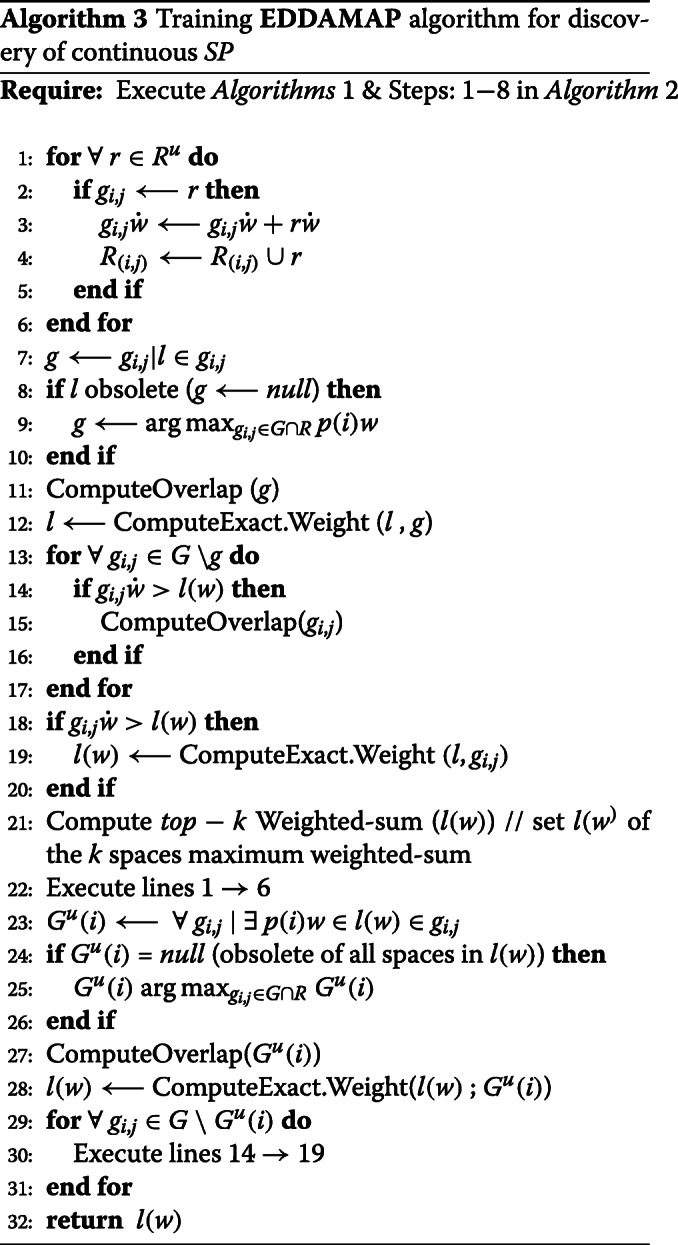


## Results

### Evaluating the algorithm

In this section, our focus is to produce and evaluate the results of the algorithm based on experimental observations. Therefore, we present the construction of the proposed algorithm to localise, recognise and monitor the *AP*. Furthermore, we discuss the performance of the training algorithm and produce the final algorithmic pseudo-code. Also, we implemented the EDDAMAP algorithm through rigorous simulations to attest to its validity in terms of accuracy and query processing speed by considering the efficiency in monitoring the *AP* alongside with the closely related G2 algorithm.

**Fig. 8:**
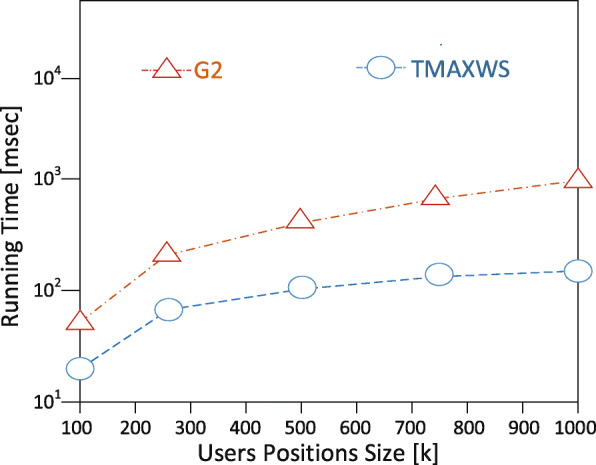
A graph of running time against position size. Showing the impact of *n* as amount of position data available for execution

**Fig. 9:**
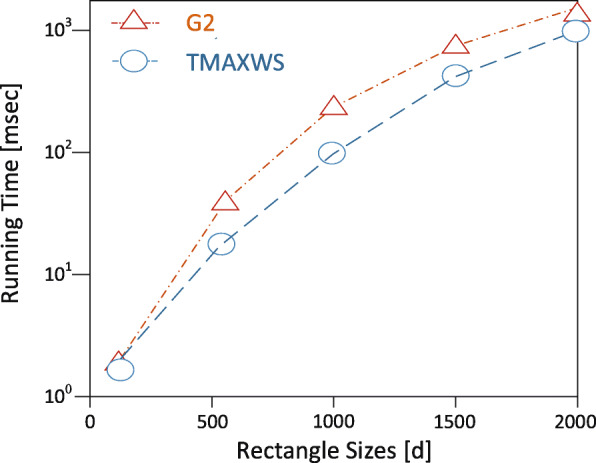
A graph of running time against rectangle sizes. Showing the impact of *d* as the increasing size of location data

**Fig. 10:**
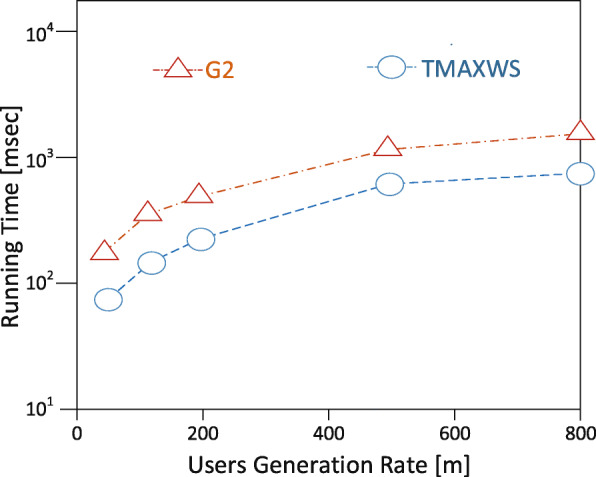
A graph of running time against user generation rate. Showing the impact of *m* as the rate of increasing position data

**Fig. 11:**
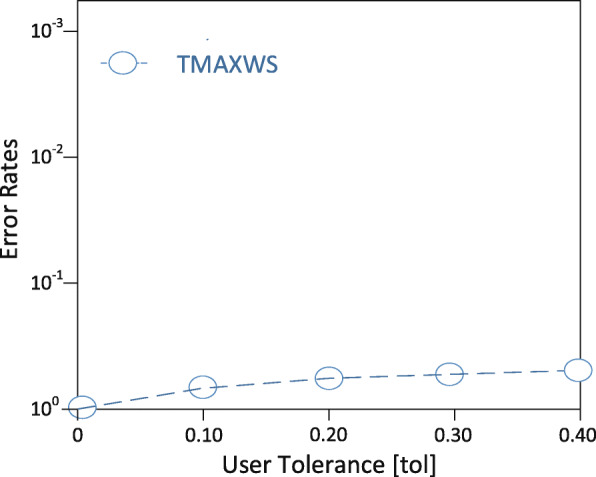
A graph of error rates against user tolerance. Showing the impact of varying user tolerance *tol* for an approximate solution

**Fig. 12:**
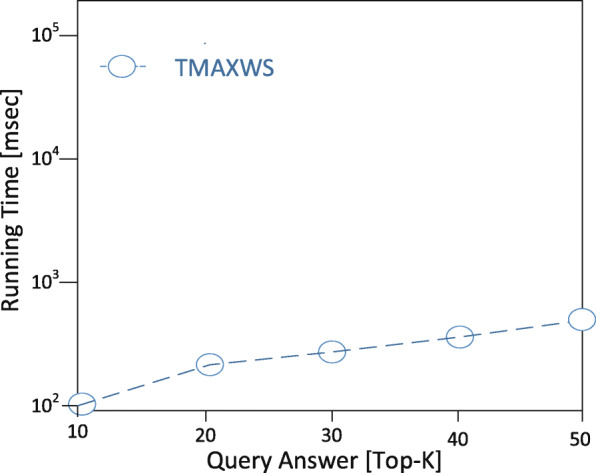
A graph of running time against query answer. Showing the impact of *top*−*k* query results

**Experimental setup** The EDDAMAP algorithm is implemented on a personal computer (PC) install with a Windows 10 operating system. The specification of the PC includes Intel Core i5 CPU 4.1 GHz and 8.00 GB RAM. Coding is done with Python and all the simulations are done using the Pycharm community version 2.7 software. In the experimentation, we mount scientific software and packages such as Scikit library, Numpy, Microsoft Visio, etc. Nonetheless, it is anticipated that a PC with a higher specification will relatively speed up the execution of the algorithm and produce a fast query result. The training algorithm is deployed and tested with a GPS-based real-world dataset known as GeoLife [35]. An ordinary mobile user is emulated as an Asymptomatic Patient (i.e. *AP*) who stays in a neighbourhood within a specified community. The *AP* is considered as performing routine activities such as exercising, shopping, hiking, sight-seeing, visit a place of interest and so on [6,36]. In our model, together with the *AP*, we try to monitor the neighbourhood which is denoted as a rectangle *r* as the specific location. And that of the community is denoted as *R* representing the general location under monitoring. The default size of the rectangle is 1000 x 1000 metres squared and that is relatively a very short range to maintain the position accuracy upon executing some query solutions. We define the other parameters in Table 2 and present the graphical results of the simulation in Figs. 8, 9, 10, 11 and 12. The legends of the graphs show the G2 and EDDAMAP algorithms as *G*2 and *TMAXWS* respectively. Note that, monitoring the MaxRS and the related problems are first introduced by Amagata and Hara [9]. Hence, to the best of our knowledge, there are no existing algorithms which can specifically and/or directly handle the MaxRS monitoring problem. Therefore, in this present study, we evaluate our proposed EDDAMAP algorithm and compare it with the G2 algorithm only for being a sufficient incremental approach.

**Table 2:** Experimental parameters

**Param**	**Default**	**Variables**	**Indicators**
*n*	500	100; 250; 500; 750; 1000	No. of position
*d*	1000	100; 500;1000; 1500; 2000	Rectangle sizes
*m*	100	50; 100; 200; 500; 800	Generation rate
*tol*	-	0; 0.10; 0.20; 0.30; 0.40	User tolerance
*k*	-	10; 20; 30; 40; 50	Query answers

**Dataset** The GeoLife dataset is a continuous-generated GPS-based trajectory dataset. We used GeoLife dataset version 1.3 and it is collected by 182 users and/or GPS loggers in more than five years, and thus from April 2007 to August 2012. The trajectories are recorded by different GPS loggers and mobile phones and sampling rates are in varying degree. The densely logged trajectories in a location represent 91.5 per cent in every 1 to 5 seconds and/or every 5 to 10 meters per point. Therefore, the dataset has a broad range of outdoor movements of users such as the usual travels to their residence and workplace. Also, it consists of the activities of the users such as a visit to recreational centres, restaurants, etc. Furthermore, the trajectories are represented by sequences of points with a time-stamp, where each of the points contains latitude, longitude and altitude records of the users. However, the altitude is not relevant in our trajectory analysis and hence it is discarded. The number of trajectories is about 17,621 points with a total distance and duration of about 1,292,951 kilometres and 50,176 hours respectively. A reasonable sample of the dataset is used to validate the test for mobility pattern mining of the users and activity recognition with our proposed technique and the related G2 algorithm, and that is due to the larger size and complexities of the dataset.

And thus, the objects (or users) present in the dataset existed over a wider range. Hence, most of the object points, if not all that existed and/or appeared in very sparse areas have to be removed (or ignored). For that matter, we focused on those object points close to the centre of the dataset. The cardinality of the dataset is about 6,134,477 points, and hence the sorting of the objects in the dataset is done based on the time in which they are generated. Consequently, the range of each coordinate is normalized to [0, 100000000] value, whereby the weight of a given object point is randomly chosen from a range of (0, 1000] value. The dataset is considered as a good choice for the testing and evaluation with the algorithms and thus because the proposed technique is aimed at the natural properties of monitoring continuous moving objects in diverse locations. This is because, the dataset is not only widely distributed in about 30 cities of the Peoples Republic of China, but it includes data from the USA and Europe. Furthermore, considering the trajectory pattern that exists in the dataset, its mining and analysis fit well with the aim of our proposed technique. Moreover, the chosen sample dataset includes the number of points that are generated in a location where the user travelled and/or visited. Thereby, a reasonable sample of the object points in the dataset is used to represent a user. Therefore, the user is emulated as an ordinary mobile phone user and/or an asymptomatic patient denoted as *AP*. GeoLife dataset is a publicly available open-source dataset that is provided by Microsoft Research Asia in Beijing. The details on how to obtain the version 1.3 can be found in the reference [35]. Further details on the GeoLife dataset, including the distribution of the number of trajectories of each user, can be obtained from the original researches [37,38]. 

Furthermore, we discuss the performance of the proposed EDDAMAP algorithm together with the key related G2 algorithm [9]. Consequently, we simulate both algorithms and present their results as shown in Figs. 8, 9, and 10. The G2 algorithm consists of a general framework for monitoring objects in spatial data streams. However, it is noticed that moving objects exhibit a continuous change in their locations over time. For that matter, the MaxRS query solution for a certain time instant will not be a solution at another time instant. Therefore, we present the proposed EDDAMAP algorithm which possesses continuous monitoring property to handle the evolving data streams environment and to serve also as an improved version of the G2 algorithm. In that regards, the proposed technique can specifically be incorporated into the public health information systems to improve the efficiency challenges that affect patient monitoring systems. As a result of the massive size of the dataset and the computing resources, the test for requirement of the proposed EDDAMAP algorithm has considered the execution time and the space complexities only. Therefore, the algorithms are designed to benefit from the novelty of maximised range-sum problem and further apply to provide a solution to the monitoring problems in general. Heuristically, we can propose that our grid index data structure as provided in Fig. 7 is relatively proven efficient for monitoring naturally occurring phenomena such as location monitoring [24], whereby related approaches suffer from overuse of resources and under-explored design techniques as they usually do not consider the natural dynamics of resources mobility, and for that matter continuous streaming object databases. In this paper, we adopt the parameters *n*, *d* and *m* as shown in Table 2 for the overall evaluation by varying them accordingly as per the features of the position of the user in the dataset. The average computation time is measured for each of the parameters and the overall simulation results are presented in Figs. 8, 9, 10, 11, and 12. Moreover, in the experiment, the impact of the number of spatial positions denoted as *n* in Table 2 (i.e. representing the *POIs* of the *AP*) has been tested. It is observed that the computation time for finding each position data increased linearly with increased in position data. This resulted in several overlaps, whereby the OverlapComputation(.) and ExactWeightComputation(.) functions heavily overloaded on execution. This implies that the larger the size of the monitoring location, the more time is needed to produce all the position data to identify the *POIs*. On the other hand, an increase in the size of the monitoring location affected the speed of generating the *POIs*, as well as executing the queries for the position of the *AP* in real-time. This is shown in the graph of running time against user spatial positions size in Fig. 8. The impact of rectangle size denoted as *d* in Table 2 is tested. This represents the monitoring location which stands for the neighbourhood and/or community of the *AP*. We observed a skewed distribution, as well as more overlaps in the rectangle as the size increased. As a result, we observed a corresponding increase in the running time of the algorithms. Therefore, it is suggested that, the user should appropriately monitor a sizeable location as possible based on the available resources of the monitoring system. The simulation result which testifies this phenomenon is presented as a graph of running time against user rectangle size as shown in Fig. 9. Again, the impact of the rate of generating position data with time which is denoted as *m* in Table 2 is tested. Here, we generated less than 50 position data as object points per second, and hence the rate has to be increased further to examine the execution time and space complexities of the algorithms. The simulation result that testifies this phenomenon is presented as a graph of running time against the user generation rate as shown in Fig. 10. Furthermore, the impact of user tolerance which is denoted as *tol* in Table 2 is evaluated by using the proposed EDDAMAP algorithm. This is to observe the error-rate and computation time when a user is executing the queries. Therefore, to validate the user tolerance denoted as *tol*, the query time is approximated based on the user preference. Consequently, the running time is seen as indirectly proportional to *tol* which suggested and/or confirmed that, there is always a trade-off between the efficiency of query processing and the quality of the query results. This also means that in a practical case the user can adjust the quality of the query results as per the expected outcome of the stay place of the user. Again, the outcome of this phenomenon is presented as a graph of running time against user tolerance as shown in Fig. 11. Also, the impact of generating a *top*−*k* (i.e. representing the best *POIs*) position data is evaluated on the average computation and/or execution time per space that is required to update the results of the query. Here, it is observed that upon increasing the *top*−*k*, the execution time proportionately increased. This suggests that the *top*−*k* query is directly proportional to the execution time per the search space, and thus when we need to obtain a new position as the *POIs*. We present the results of the process as a graph of running time against *top*−*k* position data as shown in Fig. 12. Therefore, the outcome of the increase in the query execution time suggests that the user does not need to examine the exact query solution and/or results (i.e. identify the exact *SP*). This will limit the time and space complexities so far as practical application is concerned. Conversely, it is appropriate for the user to approximate the query results and that should be done based on marginal error-rate which is also known as user-tolerance *tol* or preference value. Furthermore, when the user knows the approximated *SP* that will be enough to an informed decision in practice, and as to whether the *AP* will have the chance to spread the disease or not. Moreover, it is evidenced that approximation is preferable to that of the exact solution when dealing with real-world applications and/or databases dedicated to streaming objects.

In summary, in this section, we provide the initial evaluation and discuss the proposed EDDAMAP technique and/or algorithm based on complexities of time and space only. Upon executing a query, the outcome of the simulation validates the proposed EDDAMAP technique as having substantially improved the efficiency of monitoring continuous outdoor mobile patient such as the *AP* in a vicinity. Furthermore, the proposed technique is confirmed as an improved solution for the continuous maximising range-sum problem by comparing it to the key related G2 algorithm. However, some work remain untouched in the present study, such as a test for the scalability and performance with some other related query techniques. Nonetheless, this comparative study is reserved as future work and hence it is open for investigation. The objective of evaluating the algorithm, in terms of the processing time is to validate its efficiency in acquiring a continuously moving object data and to process queries about the present location of objects (e.g. asymptomatic patient) and/or obtaining the position of the object in real-time. The other aspect of the requirement testing includes the error-rate when querying for location and position of objects in the continuous moving object database. Considering the test for the error-rate, the G2 algorithm does not fall into the category of continuous query processing and hence cannot be compared with our algorithm. The objective of using only the real-world data aside the limited resources employed for the study is to attest to the natural occurring property of our algorithm in which the behaviour of the asymptomatic patient is regarded as a key contributing factor to attest to the natural mobility instances and patterns that the asymptomatic patient will have to exhibit along with its travel. Moreover, the overall validation of our proposed algorithm is based on the accuracy test. The accuracy is verified via simulation by taking into consideration the test for the error-rate when querying the present position and/or location of the asymptomatic patient as previously discussed.

## Conclusions

In this paper, we address a maximising range-sum problem and relate that to the challenges of monitoring the travels of an outdoor mobile asymptomatic patient and its activities in a community. As a contribution, we further establish an EDDAMAP technique and/or algorithm as a solution to the continuous moving object monitoring problem in general. Our proposed technique determines the locations of a given rectangle denoted as *R* to represent a community. Such that the number of positions of the outdoor mobile asymptomatic patient that can be obtained from a GPS-based trajectory records will be maximised in *R*. We derive another specific location denoted as *r* for a sub-location to represent a neighbourhood in *R*. The *r* covers the positions of the asymptomatic patient, and our algorithm is aimed to locate the centroid of *r* as the place of interests denoted as *POIs* and/or stay place denoted as *SP*, depending on the time and nature of activities of the asymptomatic patient in that location and/or neighbourhood. In contrast to the original MaxRS problem, we look at continuous MaxRS query where the query results change over time as the asymptomatic patient keeps moving and/or visiting interesting places over time. To match up with the query processing time, we design a grid-based index data structure as shown in Fig. 7 to ensure efficient continuous monitoring of a change in position and/or location of the asymptomatic patient in real-time. Furthermore, techniques such as branch and bound, current object localisation and recognition and graph theory are adopted to ensure the validity of the proposed design technique and/or algorithm. Our experiment using real-life dataset shows that the proposed technique heuristically will enhance the accuracy and efficiency in monitoring mobile objects in general. Furthermore, it will also enable a significant speed in the overall computation time per space for the upper bound with respect to the location under searching or monitoring. This will help satisfy the time and space complexities problems by considering the location and/or position in the continuous moving object database. The proposed EDDAMAP technique and/or algorithm will promote the discovery of endemic zones as far as a pandemic is concerned via the naturally occurring Spatio-temporal activities of an asymptomatic patient. Nonetheless, the public healthcare setting can also apply the proposed algorithm to facilitate the design and management of multi-modal health information systems such as patient monitoring system applications to curtail global pandemic.

In future work, we will look at the enhancement of the theoretical analysis. We will also extend the practical applications of the established technique to more multi-modal monitoring systems. Thus, we aim to ensure that the proposed algorithm can handle extra computation and storage by considering the tight upper-bound of the maximised weighted range-sum of the *POIs* in every location. In this manner, the EDDAMAP algorithm will be able to handle queries of multiple instances to enhance its scalability and performance. It is our extra hope to intensify this burgeoning trajectory data mining technique in the research community and the industry.

## Data Availability

The datasets used and/or analysed during the current study are publicly available from Microsoft Asia Beijing website or request as to how to access the data should be sent to the corresponding author on reasonable request.

## Abbreviations

AP: Asymptomatic patient
EDDAMAP: Efficient data-dependent approach for monitoring asymptomatic patient
PMS: patient monitoring system
GPS: Global positioning system
POIs: Place/Point of interests
SP: Stay place
SPL: Single-point location
MPL: Single-point location
SVM: Support-vector machine
G2: Graph in grid index
TMaxWS: Trajectory maximised-weighted sum
PC: Personal computer

## References

[1] Crepaldi NY, de Lima IB, Vicentine FB, Rodrigues LML, Sanches TLM, Ruffino-Netto A, Alves D, Rijo RPCL (2018). Towards a clinical trial protocol to evaluate health information systems: evaluation of a computerized system for monitoring tuberculosis from a patient perspective in Brazil. J Med Syst.

[2] Hassan MM, Huda S, Uddin MZ, Almogren A, Alrubaian M (2018). Human activity recognition from body sensor data using deep learning. J Med Syst.

[3] Gonzalez MC, Hidalgo CA, Barabasi AL (2008). Understanding individual human mobility patterns. Nature.

[4] Beltrame T, Amelard R, Wong A, Hughson RL (2018). Extracting aerobic system dynamics during unsupervised activities of daily living using wearable sensor machine learning models. J Appl Physiol.

[5] Nogueira P, Urbano J, Reis LP, Cardoso HL, Silva DC, Rocha AP, Gonçalves J, Faria BM (2018). A review of commercial and medical-grade physiological monitoring devices for biofeedback-assisted quality of life improvement studies. J Med Syst.

[6] Adu-Gyamfi D, Zhou F, Zhang F, Kibiwott KP, Tattrah VD. Real-time monitoring of mobile user using trajectory data mining* In: Sengodan T, et al, editors. 2019 Third IEEE International Conference on Electrical, Computer and Communication Technologies. IEEE: 2019. p. 1–8.

[7] El-Sappagh S, Ali F, Hendawi A, Jang J-H, Kwak K-S (2019). A mobile health monitoring-and-treatment system based on integration of the SSN sensor ontology and the HL7 FHIR standard. BMC Med Inform Decis Mak.

[8] Esposito M, Minutolo A, Megna R, Forastiere M, Magliulo M, De Pietro G (2018). A smart mobile, self-configuring, context-aware architecture for personal health monitoring. Eng Appl Artif Intell.

[9] Amagata D, Hara T (2017). A general framework for MaxRS and MaxCRS monitoring in spatial data streams. ACM Trans Spat Algorithm Syst.

[10] Zhang P, Deng M, Shi Y, Zhao L (2017). Detecting hotspots of urban residents’ behaviours based on spatio-temporal clustering techniques. GeoJournal.

[11] Qiu W, Bandura A. GPS trace mining for discovering behaviour patterns. In: 2015 International Conference on Intelligent Environments: 2015. p. 65–72, IEEE.

[12] Hussain MM, Trajcevski G, Islam KA, Ali ME. Towards efficient maintenance of continuous maxrs query for trajectories. In: 20th International Conference on Extending Database Technology (EDBT). OpenProceedings.org; 2017. p. 402–13.

[13] Zheng VW, Zheng Y, Xie X, Yang Q (2012). Towards mobile intelligence: Learning from GPS history data for collaborative recommendation. Artif Intell.

[14] Or C, Tong E, Tan J, Chan S (2018). Exploring factors affecting voluntary adoption of electronic medical records among physicians and clinical assistants of small or solo private general practice clinics. J Med Syst.

[15] Rajkumar S, Muttan S, Sapthagirivasan V, Jaya V, Vignesh SS (2018). Development of improved software intelligent system for audiological solutions. J Med Syst.

[16] Singh D, Merdivan E, Psychoula I, Kropf J, Hanke S, Geist M, Holzinger A, Kieseberg P, Tjoa A, Weippl E (2017). Human activity recognition using recurrent neural networks. Machine Learning and Knowledge Extraction. CD-MAKE 2017. Lecture Notes in Computer Science.

[17] Wu R, Luo G, Shao J, Tian L, Peng C (2018). Location prediction on trajectory data: A review. Big Data Min Analytics.

[18] Moral-Munoz JA, Esteban-Moreno B, Herrera-Viedma E, Cobo MJ, Perez IJ (2018). Smartphone applications to perform body balance assessment: a standardized review. J Med Syst.

[19] Lee S, Holzinger A. Knowledge discovery from complex high dimensional data In: Michaelis S, Piatkowski N, Stolpe M, editors. Solving Large Scale Learning Tasks: Challenges and Algorithms. Springer Lecture Notes in Computer Science: 2016. p. 148–67.

[20] Pawar P, Jones V, van Beijnum BJF, Hermens H (2012). A framework for the comparison of mobile patient monitoring systems. J Biomed Inform.

[21] Yu Z (2015). Trajectory data mining: An overview. ACM Trans Intell Syst Tech.

[22] Yeh T, Lee JJ, Darrell T. Fast concurrent object localization and recognition. In: 2009 IEEE Conference on Computer Vision and Pattern Recognition: 2009. p. 280–7.

[23] Lettich F, Orlando S, Silvestri C. Processing streams of spatial k-NN queries and position updates on manycore GPUs. In: Proceedings of the 23rd SIGSPATIAL International Conference on Advances in Geographic Information Systems: 2015. p. 1–10. SIGSPATIAL 15.

[24] Costa C, Chatzimilioudis G, Zeinalipour-Yazti D, Mokbel MF. Efficient exploration of telco big data with compression and decaying. In: 2017 IEEE 33rd International Conference on Data Engineering. IEEE Computer Society: 2017. p. 1332–43.

[25] Yang Y, Du B, Jiang X. A human trajectory estimate based on individual mobility pattern library. In: 2013 IEEE International Conference on Green Computing and Communications and IEEE Internet of Things and IEEE Cyber, Physical and Social Computing. IEEE: 2013. p. 1181–5.

[26] Giannotti F, Nanni M, Pedreschi D, Renso C, Trasarti R. Mining mobility behavior from trajectory data. In: 2009 International Conference on Computational Science and Engineering. IEEE: 2009. p. 948–51.

[27] Adu-Gyamfi D, Zhang F, Zhou F. Finding influential location via user mobility and trajectory In: Sengodan T, et al, editors. Advances in Electrical and Computer Technologies. Lecture Notes in Electrical Engineering, Springer Nature Singapore Pte Ltd.: 2020. p. 233–46.

[28] Lampert CH, Blaschko MB, Hofmann T. Beyond sliding windows: Object localization by efficient subwindow search. In: Proceedings of the 2008 IEEE Conference on Computer Vision and Pattern Recognition: 2008. p. 1–8.

[29] Hu H, Zhai X, Wang M, Hu G (2019). Graph analysis of network flow connectivity behaviors. Turk J Electr Eng Comput Sci.

[30] Bakalov P, Tsotras VJ, Nittel S, Labrinidis A, Stefanidis A (2008). Continuous spatiotemporal trajectory joins. International Conference on GeoSensor Networks.

[31] Feng Y, Ji M, Xiao J, Yang X, Zhang JJ, Zhuang Y, Li X (2015). Mining spatial-temporal patterns and structural sparsity for human motion data denoising. IEEE Trans Cybernet.

[32] Li F, Long X, Du S, Zhang J, Liu Z, Li M, Gui Z, Yu H. Analyzing campus mobility patterns of college students by using GPS trajectory data and graph based approach. In: 2015 23rd International Conference on Geoinformatics: 2015. p. 1–5.

[33] Ebadi N, Kang JE, Hasan S (2017). Constructing activity-mobility trajectories of college students based on smart card transaction data. Int J Transp Sci Tech.

[34] Cho E, Myers SA, Leskovec J. Friendship and mobility: User movement in location-based social networks. In: 17th ACM SIGKDD International Conference on Knowledge Discovery and Data Mining: 2011. p. 1082–90.

[35] Zheng Y, Xie X, Ma W-Y (2010). GeoLife: A collaborative social networking service among user, location and trajectory. IEEE Data Eng Bull.

[36] Adu-Gyamfi D, Zhang F (2020). Towards derail of global pandemics via patient trajectory. J Appl Sci Eng.

[37] Zheng Y, Zhang L, Xie X, Ma W-Y. Mining interesting locations and travel sequences from GPS trajectories. In: International Conference on World Wild Web (WWW). ACM Press: 2009. p. 791–800.

[38] Zheng Y, Li Q, Chen Y, Xie X, Ma W-Y. Understanding mobility based on GPS data. In: Proceedings of the 10th international conference on Ubiquitous computing. ACM Press: 2008. p. 312–21.

